# Education in the placement of ultrasound-guided peripheral venous catheters: a systematic review

**DOI:** 10.1186/s13049-021-00897-z

**Published:** 2021-06-27

**Authors:** Rasmus Jørgensen, Christian B. Laursen, Lars Konge, Pia Iben Pietersen

**Affiliations:** 1 Department of Respiratory Medicine, Odense University Hospital, Sdr. Boulevard 29, 5000 Odense, Denmark; 2 Regional Center for Technical Simulation, Region of Southern Denmark, 5000 Odense, Denmark; 3 Department of Clinical Research, Faculty of Health Science, University of Southern Denmark, Odense, Denmark; 4 Copenhagen Academy for Medical Education and Simulation, University of Copenhagen and the Capital Region of Denmark, Copenhagen, Denmark

## Abstract

**Background:**

Placing a peripheral vein catheter can be challenging due to several factors, but using ultrasound as guidance increases the success rate. The purpose of this review is to investigate the knowledge already existing within the field of education in ultrasound-guided peripheral vein catheter placement and explore the efficacy and clinical impact of different types of education.

**Methods:**

In accordance with PRISMA-guidelines, a systematic search was performed using three databases (PubMed, EMBASE, CINAHL). Two reviewers screened titles and abstracts, subsequently full-text of the relevant articles. The risk of bias was assessed using the Cochrane Collaboration risk of bias assessment tool and the New Ottawa scale.

**Results:**

Of 3409 identified publications, 64 were included. The studies were different in target learners, study design, assessment tools, and outcome measures, which made direct comparison difficult. The studies addressed a possible effect of mastery learning and found e-learning and didactic classroom teaching to be equally effective.

**Conclusion:**

Current studies suggest a potential benefit of ultrasound guided USG-PVC training on success rate, procedure time, cannulation attempts, and reducing the need for subsequent CVC or PICC in adult patients. An assessment tool with proven validity of evidence to ensure competence exists and education strategies like mastery learning, e-learning, and the usage of color Doppler show promising results, but an evidence-based USG-PVC-placement training program using these strategies combined is still warranted.

**Supplementary Information:**

The online version contains supplementary material available at 10.1186/s13049-021-00897-z.

## Introduction

Peripheral vein catheters (PVC) play a crucial role in the treatment of hospitalized patients. The number of difficult intravenous access (DIVA) patients is substantial because problems occur when obesity, dehydration or hematologic diseases make traditional PVC placement difficult [1]. In these cases the clinicians can be forced to resort to a less optimal alternative.

The use of ultrasound guidance could be a solution for ensuring a PVC placement in DIVA patients. Ultrasound-guided PVC (USG-PVC) placement is a complex procedure that requires confidence in using equipment and understanding of complex imaging with the transfer of 2D pictures to a 3D world. Since ultrasound procedures, in general, are shown to be highly user-dependent it is questionable whether a novice user would be able to perform USG-PVC with no previous training [2].

An educational program using phantoms and simulation training could improve health professionals’ knowledge and confidence and thereby provide a solution for PVC placement in DIVA patients. An improvement is seen for other technical procedures like central vein catheter (CVC) placement or lumbar puncture [3, 4]. For CVC a meta-analyse showed that trainees going through a simulation program had significant lager proportion of successfully placed CVC [5]. It is not clear which type of training is better for USG-PVC, and what clinical impact implementation of an educational program will have. A systematic review might be able to clarify this, as seen in the cases of educational programs for other procedures [6, 7]. To our knowledge, no systematic review providing an overview of the already existing research on USG-PVC training has been carried out.

Therefore, we have conducted this systematic review with the aim to 1) investigate the already existing knowledge within the field, 2) explore which type of education seems to be the most effective, and 3) assess the potential clinical impact of an education program.

## Material and method

This systematic review was performed in accordance with the Preferred Reporting Items for Systematic Reviews and Meta-Analyses (PRISMA) [8].

### Search and study selection

In collaboration with a research librarian, a systematic literature search of three databases (CINAHL, PubMed, EMBASE) was conducted using relevant search terms related to USG-PVC placement. Full search strategies can be found in Additional file 1. The first search was conducted on December 10, 2017, and a final search was conducted on February 13, 2021, to ensure the results being up to date. Two authors (RJ, PIP) independently screened all articles for eligibility based on titles and abstracts. Relevant studies were then reviewed independently as full-text for final inclusion. A third investigator (CBL) resolved any inclusion disagreements. Finally, a hand search through the reference lists of included articles was conducted and expert recommendations were screened for inclusion as well.

### Eligibility criteria

Original research articles were included if both of the following were present:

- Assessment of educational process or simulation training in context of USG-PVC placement
- Scandinavian, English, or German language

### Exclusion criteria

Articles were excluded if one or more of the following were present

- Conference abstracts, explanatory articles, teaching books and expert opinions
- Articles not involving USG-PVC placement and only involving CVC or peripherally inserted central catheter (PICC)

No restrictions were made for either patient population, or the type of health personnel performing the USG-PVC procedure.

### Data extraction

The following data were extracted from each article: Study type, characteristics of the study population, characteristics of the patient population, educational program, technique of USG-PVC placement, and results.

### Risk of bias assessment

A modified risk of bias evaluation based on the criteria of the Cochrane Collaboration risk of bias assessment tool was performed on all included studies [9]. Non-randomized studies were assessed using the Newcastle-Ottawa Scale bias tool [10].

### Data synthesis

Due to the wide aim of the research question and a significant heterogeneity in study designs, a meta-analysis could not be performed and a descriptive synthesis and approach was therefore applied.

## Results

**Fig. 1:**
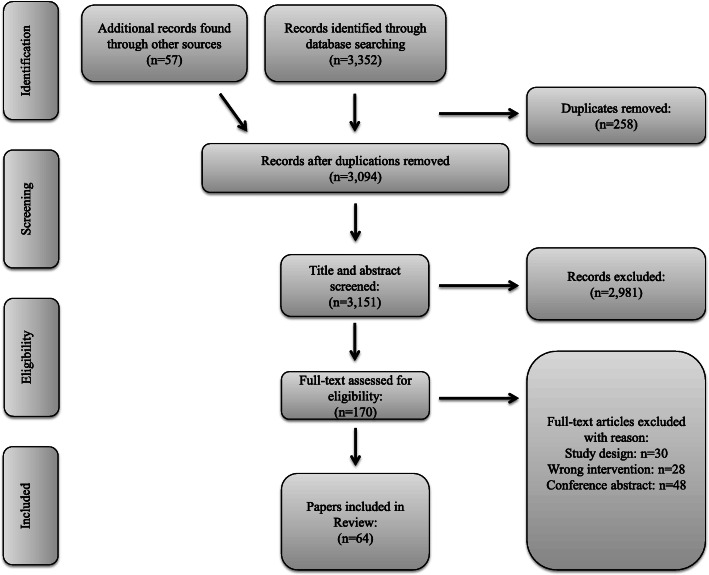
Flowchart of search strategy and selection process based on the preferred reporting items for systematic reviews and meta-analyses (PRISMA)

The initial search performed on December 10th, 2017, generated a total of 2207 publications, and the additional search on February 13th, 2021, added to a total number of 3352. Additional 57 articles were full-text screened after being identified through hand search of reference lists. Of the total of 3409 articles 170 were full-text screened. The full text screening resulted in a total of 64 included articles. Study flow of article identification and selection is shown in Fig. 1.

Sixty-one studies included an educational program, two were Delphi studies, and one was a prospective validity study of an assessment tool.

### Assessment of need

No needs-assessment studies were found, but a few studies evaluated participants’ impression of skills and the self-defined benefits from participation after completing the education [11–15]. Participants found the education program helpful and felt able to place PVC in patients where it would otherwise not have been possible before education. The participants estimated a need for USG-PVC to be approximately six to seven patient cases every week. They felt an overall improvement of personal skills and considered it possible to acquire the necessary skills for ultrasound guided PVC placement through the utilized educational program [12, 14, 15].

### Curriculum

In most of the included studies the curriculum was poorly described but generally included; ultrasound physics, knobology, probe selection, ultrasonic vascular and nerve recognition, preparation for USG-PVC placement, and complications (Additional file 3).

### Educational methods and technique

In six studies the USG-PVC education was a part of a general ultrasound curriculum [16–21]. These educational programs included other ultrasound examinations like echocardiography and focused lung ultrasound among other techniques. The lengths of these general educational programs were between 15 to 20 h or a full rotation at an emergency ultrasound department. Five studies included scheduled follow-up with either more than 100 scans (*n* = 3) or at least 2 weeks of emergency medicine rotation to ensure competency (*n* = 5) [16, 18–21].

In studies only training the USG-PVC, the training comprised of a combination of didactic and hands-on sessions, including education on live models for normal vascular anatomy and cannulation on phantoms (Additional file 3). Fourteen educational programs included video material of the procedure [12–14, 22–32] and five included live demonstrations of the procedure [22, 23, 29, 33, 34]. Inter-study duration variations in the training sessions were seen from the shortest of 5 min to the longest of 9 h [35, 36]. In general, the duration of the training was between two to 4 h (Additional file 3).

Three studies were based on the mastery learning approach and two other studies used a similar approach [15, 28–31]. It took the participants less than 1 h of training before meeting the requirement for passing in two studies [29, 31]. A fixed curriculum time limit was used in two of the five studies, and in these two studies participants failing the first assessment attempt only required additional 15–60 min of training to pass in a second attempt [28, 30]. One study showed that extra training after meeting mastery criteria did not improve the participants’ performance [31].

E-learning, test-enhanced learning, and the composition of phantoms for training were evaluated [37–39]. None of these were found to have a positive or negative effect on learning competencies. Teaching participants to use colour Doppler for what is called “twinkle artifact” showed an improvement compared with only using cross-section view [40]. Two studies found a relatively steep learning curve after initial training, and that after four to nine real world attempts a nurse’s probability of success was over 70% on average [41, 42]. Likewise, results were seen in paediatric patients with a success rate of 67% after ten supervised attempts, and an increase after ten additional unsupervised attempts to 83% [27]. After ten successful attempts Ault et al. showed that the participants’ learning curve flattened [43].

### Participants and training objects

The participants in the included studies varied from doctors/physicians (*n* = 12), nurses (*n* = 24), emergency department technicians (ED-technicians) (*n* = 3), nurse students (*n* = 2), medical students (*n* = 7) and in one study it was unclear. Additional 13 studies included more than one of the groups mentioned above. In-depth information about education participants can be found in Additional file 3.

USG-PVC methods and education were evaluated through different parameters like success rate, time, attempts, and participant rating mainly through Likert-scales. Sixty-one studies assessed the learners using mainly, two objects; either phantoms (*n* = 17) or living subjects (*n* = 38). The remaining studies did not use any evaluation objects and only evaluated through participant feedback (*n* = 6). The living subjects could be patients (*n* = 36) and some of the studies specified the type of patient. Adult patients were used in 20 studies, whereas children were used in five studies. Fifteen studies included only patients specified as DIVA patients. In other studies, the living subjects were healthy persons (*n* = 2). The phantoms used in studies were commercially available (*n* = 10) [13, 23, 34, 35, 38, 40, 44–47], homemade (*n* = 5) [26, 37, 39, 48, 49], or a mixture of both types of phantoms [50]. One study did not specify the type of phantom they used [51]. In-depth information about test subject and phantom distribution can be found in Additional file 3.

### Assessment of competence

Assessing the competence of participants through assessment tools was done in seven studies [22, 28, 30, 31, 47, 52, 53]. In all seven studies the used assessment tool was a checklist. Good et al. evaluate if it is meaningful to use motion analysis as a tool for assessing competency for USG-PVC placement [47]. They did this by comparing a group of nurses’ motion analysis before and after USG-PIV education and comparing their improvement to the result of experts’ motion analyses. Good et al. found that 17 out of 21 nurses obtained expert proficiency in at least one of six motion-analysis metrics after education.

Two competence assessment tools were identified: one as a checklist of competence and one as a rating scale for competencies [54, 55]. Validity evidence was only explored for the rating scale [56].

### USG-PVC techniques

Different types of USG-PVC techniques were evaluated in studies carried out on patients, phantoms, or both [12, 13, 18, 23, 25, 34, 35, 44–48, 57–59]. When evaluating the effect of long-axis view or short-axis view, most studies found no difference. Both the long-axis and the short-axis method were found to be better than the oblique method [35]. The differences between using the dominant or non-dominant hand for probe handling were evaluated in one study, that found the dominant to be superior [23]. Results comparing one versus two-person techniques were inconclusive [13, 58] and the implementation of guidance-markers on the screen only showed an effect when used by nurses [48].

Success rate for cannulation, number of attempts or time were the most frequently used variables for evaluating the clinical effects of education and implementation of USG-PVC placement. Likert-scales for pain and participant satisfaction were used as well. The most relevant outcomes are listed in the data-extraction sheet (Additional file 3).

### Clinical impact

Traditional PVC placement without ultrasound guidance was primarily used as control. In some studies the traditional technique was specified as being anatomically guided. Nine out of ten studies found a significant difference in time, attempts, or cannulation success rate favouring ultrasound guidance compared to conventional methods on patients [16, 19, 32, 52, 60–64], whereas the remaining one did not find any significant difference [65]. The effect in these studies was only seen on DIVA patients. A decrease in the need for PICC or CVC was seen in several studies after the implementation of education in ultrasound guidance for PVC placement [21, 29, 62, 66, 67].

Lastly, two studies compared different health-professional groups and found no significant difference in success rate between nurses, physicians, or ED-technicians [25, 68].

Only outcomes assessed relevant to the aim of this study are included in this results section. For a full overview of outcomes see the extended data-extraction sheet in Additional file 3.

### Risk of bias

The Cochrane risk-of-bias tool was used to evaluate all studies included and 33 studies were also evaluated through the Newcastle-Ottawa scale, but the heterogeneity of the included studies made the risk of bias hard to address and compare. The risk-of-bias assessments are shown in Tables 1 and 2.

**Table 1:** Scores of the Cochrane tool of Bias

	Selection bias		Performance bias	Detection bias		Attrition bias		Reporting bias		Other bias
	Random sequence generation	Allocation concealment	Blinding of participants and personnel	Blinding of outcome assessment		Incomplete outcome data		Selective reporting		Anything else, ideally pre-specified
Bahl [32]	Low	Low	/ (High)	High		Low		? / (High)		Low
Bauman [61]	/	/	/ (High)	High		Low		High		High
Breslin [15]	/	/	/ (High)	High		High		High		Low
Desai [20]	/	/	/ (High)	High		Low		High		Low
Duran-Gehring [22]	/	/	/ (High)	High		Low		Low		Low
Feinsmith [52]	/	/	/ (High)	High		High		High		Low
Vitto [51]	? (High)	? (High)	/ (High)	High		High		High		High
Moore [53]	/	/	/ (High)	High		High		High		High
Oakley [60]	/	/	/ (High)	High		Low		High		High
Bair [69]	Low	Low	/ (High)	High		Low		Low		Low
Osborn [11]	? (High)	? (High)	/ (High)	High		Low		High		Low
Costantino [16]	High		/ (High)	High		High		High		Low
Ault [43]	/	/	/ (High)	High		High		High		Low
Fürst [49]	/	/	/ (High)	High		Low		High		High
Clemmensen [34]	Low	High	/ (High)	Low		Low		High		Low
Blaivas [57]	High	High	/ (High)	High		Low		High		Low
Brannam [24]	/	/	/ (High)	High		High		High		Low
Carter [68]	Low	High	/ (High)	High		High		High		Low
Chinnock [58]	/	/	/ (High)	High		Low		High		Low
Davis [39]	High	High	/ (High)	High		Low		High		High
Stolz [42]	/	/	/ (High)	High		High		High		Low
Durand-Bailloud [23]	High	High	/ (High)	High		High		High		Low
Erickson [45]	/	/	Low	Low		Low		High		Low
Griffiths [44]	Low	Low	/ (High)	High		Low		High		Low
Oliveira [25]	/	/	/ (High)	High		High		High		Low
Jung [55]	/	/	/ (High)	/						
Leung [50]	/	/	/ (High)	High		High		High		Low
Tassone [35]	Low	High	/ (High)	High		Low		Low		Low
Adhikari [14]	/	/	/ (High)	? (High)		? (High)		High		Low
Blaivas [26]	/	/	/ (High)	High		High		High		Low
Schoenfeld [70]	/	/	/ (High)	High		Low		High		Low
Edwards [12]	/	/	/ (High)	High		High		High		High
Bridey [65]	Low	High	/ (High)	High		Low		High		Low
Good [47]	/	/	/ (High)	High		High		High		Low
Sou [64]	/	/	/ (High)	High		Low		High		Low
Mahler [59]	Low	Low	/ (High)	High		Low		High		Low
Shokoohiet [21]	/	/	/ (High)	High		High		High		Low
Costantino [19]	High	High	/ (High)	High		High		High		High
Doniger [63]	Low	Low	/ (High)	High		Low		High		Low
Panebianco [18]	/	/	/ (High)	High		High		High		Low
Maiocco [66]	/	/	/ (High)	High		High		High		High
Reeves [36]	/	/	/ (High)	Low		Low		High		Low
Primdahl [54]	/	/	Low	Low		/				
Primdahl [56]	/	/	/ (High)	High		Low		High		Low
Chenkin [38]	Low	Low	/ (High)	High		High		Low		Low
Gopalasingam [67]	/	/	/ (High)	High		High		Low		Low
Salleras-Duran [17]	/	/	/ (High)	High		Low		High		Low
Stone [46]	Low	High	/ (High)	High		High		High		High
Carrie [13]	/	/	/ (High)	High		Low		High		Low
Partovi-Deilami [62]	/	/	/ (High)	High		High		High		High
Vinograd [71]	/	/	/ (High)	High		High		High		High
Dargin [72]	/	/	/ (High)	High		Low		High		High

/ = not relevant or not possible

? = Unclear

**Table 2:** Scores of the Newcastle-Ottawa Scale

Authors	Study type	Selection				Comparability*		Outcome		
		Representativeness of the exposed cohort	Selection of the non exposed cohort	Ascertainment of exposure	Demonstration that outcome of interest was not present at start of study	1) Study controls for _______ (most important factor)	1) Study controls for any additional factor	Assessment of outcome	Was follow-up long enough for outcomes to occur?	Adequacy of follow up for cohorts
Bauman [61]	Cohort study	2(★)	1(★)	1(★)	1(★)	Difficult vein access (★)	/	3	1(★)	4
Oakley [60]	Prospective observational study	3	1(★)	1(★)	2	Difficult vein access (★)	/	3	1(★)	2(★)
Feinsmith [52]	Cohort study	3	1(★)	1(★)	1(★)	Difficult vein access (★)	/	2(★)	1(★)	2(★)
Schoenfeld [70]	Prospective observational study	3	3	1(★)	2	Difficult vein access (★)	/	2(★)	1(★)	2(★)
Blaivas [26]	Prospective observational study	2(★)	/	1(★)	/	/	/	3	1(★)	2(★)
Desai [20]	Cross-sectional study	2(★)	3	1(★)	1(★)	/	/	2(★)	1(★)	3
Partovi-deilami [62]	Cohort study	3	1(★)	1(★)	1(★)	On-call access team (★)	/	2(★)	1(★)	2(★)
Carrie [13]	Cross sectional study	2(★)	/	3	1(★)	/	/	2(★)	1(★)	1(★)
Adhikari [14]	Cross sectional study	2(★)	/	1(★)	2	/	/	3	1(★)	1(★)
Oliveira [25]	Cross sectional study	3	/	1(★)	1(★)	/	/	3	1(★)	3
Erickson [45]	Prospective observational study	2(★)	1(★)	1(★)	1(★)	/	/	1(★)	1(★)	1(★)
Ault [43]	Cohort study	3	1(★)	1(★)	2	/	/	4	1(★)	1(★)
Stolz [42]	Prospective observational study	3	1(★)	1(★)	1(★)	/	/	2(★)	1(★)	1(★)
Gopala-singam [67]	Cohort study	3	1(★)	1(★)	2	Apheresis patients (★)	/	1(★)	1(★)	1(★)
Chinnock [58]	Prospective observational study	4	/	1(★)	2	/	/	4	1(★)	1(★)
Sou [64]	Cohort study	2(★)	1(★)	1(★)	2	Difficult vein access (★)	/	2(★)	1(★)	1(★)
Good [47]	Cohort study	2(★)	1(★)	1(★)	1(★)	/	/	2(★)	1(★)	1(★)
Shokoohi [21]	Cohort study	2(★)	1(★)	1(★)	2	/	/	2(★)	1(★)	2(★)
Panebianco [18]	Cohort study	3	/	1(★)	2	/	/	2(★)	1(★)	1(★)
Maiocco [66]	Observational study	3	2	1(★)	2	/	/	2(★)	1(★)	2(★)
Reeves [36]	Cohort study	2(★)	?	1(★)	2	Difficult vein access (★)	/	2(★)	1(★)	4
Salleras-duran [17]	Not stated (cross-sections study)	2(★)	1(★)	3	2	/	/	3	1(★)	1(★)
Vinograd [71]	Cohort study	3	1(★)	4	2	/	/	2(★)	2	3
Dargin [72]	Cross sectional study	1(★)	/	1(★)	2	/	/	2(★)	2	2(★)
Thorn [48]	Cohort study	1(★)	1(★)	3	2	/	/	2(★)	1(★)	1(★)
Galen [29]	Cohort/case control	1(★)	1(★)	1(★)	1(★)	/	/	2(★)	2	2(★)
Blick [73]	Observational study	2 (★)	/	1(★)	/	/	/	3	1(★)	1(★)
Anderson [41]	Observational study	1(★)	/	2(★)	2	/	/	3	1(★)	/
Batten [33]	Cohort study	3	2	2(★)	1(★)	/	No previous Us experience (★)	2(★)	1(★)	1(★)
Ballard [28]	Cohort study	1(★)	1(★)	1(★)	1(★)	/	/	2(★)	1(★)	1(★)
Amick [30]	Prospective cohort study	2(★)	1(★)	2(★)	1(★)	/	/	2(★)	1(★)	2(★)
Bortman [74]	Cohort study	3	1(★)	2(★)	1(★)	/	/	4	2	3
Kaganocskaya [75]	Quasiexperimental study (prospective cohort)	3	1(★)	1(★)	2	/	/	2(★)	1(★)	1(★)

Table 2: Risk of bias assessment Newcastle-Ottawa scale [10]

The Newcastle-Ottawa scale risk assessment is based on three different areas; selection, comparability and outcome. In each a certain amount of stars can be given. These stars represent the quality in that focus area. The maximum amount of stars a study can get is 9 spread over three categories as 4/2/3 (selection/comparability/outcome). For more information, visit the Newcastle-ottawa scales website.

## Discussion

In this systematic review of education in USG-PVC, we found that in especially the inpatient groups categorized as DIVA-patients, the implementation of an educational program resulted in a better patient-outcome. An effect that seems also to be present in children, but additional evidence is needed to confirm and clarify this. A large heterogeneity in the educational programs makes it hard to draw clear conclusions on how to construct the best curriculum for USG-PIV. Additionally, only few studies compared different educational methods and strategies. However, few points are possible draw from this review. The primary focus of many of the included studies was the clinical impact and evaluation of different techniques, this restricted the possibilities to recommend educational approaches other than which technique to train.

### Developing an educational program

The aim of medical educational programs is to provide its participants with sufficient knowledge or competences in specific areas or procedures defined through a curriculum. Ideally, the educational program should lead to a clinical improvement and ensure a minimum of competence for all participants.

Kern’s six-step model for curriculum development can be used to ensure that an education program fulfils the aim just mentioned above [76, 77]. The six steps are; problem identification and general needs-assessment, targeted needs-assessment, setting goals and measurable objectives, educational strategies, implementation, and evaluation and feedback. In this review, we use Kern’s model to evaluate and discuss the knowledge in USG-PVC education existing at the moment. The first two steps, general and targeted needs-assessment, help to define a demand for a given education and explore the current knowledge limitations within the field [78]. To clarify the extent of the curriculum broad goals and objectives are determined (step three). Assessment tools preferably based on solid evidence of validity should be used to ensure these goals. The educational program is then implemented in a clinical setting, where it is tested for its effect and impact, and lastly evaluating the whole process to chance or refine some of the five previous steps. Throughout each step, the previous steps should be evaluated and changes considered.

### Needs assessment

When assessing medical education, it is recommended to carry out a general needs-assessment on a national or international level [76, 78]. A national general needs-assessment in anaesthesiology identified the technical procedures that leading key-opinion leaders thought should be trained using simulation [79]. USG-PVC placement was mentioned as one of 30 important procedures.

Edwards et al. and Adhikari et al. investigated nurses’ perceived need for an educational program through questionnaires after completion of USG-PVC training [12, 14]. Nurses expected approximately three DIVA-patients per shift and thought that ultrasound guidance might be an aid. More than half the nurses felt that the biggest barrier for them to use USG-PVC was lack of experience and almost everyone agreed that focused training was adequate to learn this*.* The two studies addressed a local perception of need for education but in reverse order. A needs-assessment could be put in place to clarify the need for education, preferably with the possibility for generalisation.

### Goals and objectives

General goals and specific measurable objectives are important because they help define the curriculums and direct content and participant focus [76]. Benchmarks can through assessment tools as checklists or global rating scales ensure participants’ competence during and after education programs.

A lack of a defined curriculum and a rare use of any tools for ensuring competencies are seen in the included articles. Furthermore, seven of the included articles use a general US education [16–21, 71], which makes it harder to address the effect of the USG-PVC training and open up for the possibility for transfer of skills. Ahern et al. investigated general ultrasound educations in America and found a lack of assessment tools and lack of specific curricula [80]. However, there seems to be a general consensus in the included articles regarding which topics are important e.g. ultrasound and machine understanding, knobology, probe handling, ultrasound picture recognition and ultrasound cannulation technique.

The Delphi-method can help define the extent of a curriculum together with clearly defined goals and sub-goals [81]. Messick’s framework can then be used to ensure the assessment tool’s capability to measure what it is supposed to measure [82]. The framework describes five sources of validity in experimental data consist of five groups; content, response process, internal structure, relationship to other variables, and consequences. Each source of validity can demand varying attention depending on the curriculum assessed [83].

Two assessment tools were found; Jung’s 16 items checklist and Primdahl’s rating scale, both developed through the Delphi-method. Only Primdahl et al. explored the validity evidence of the assessment tool using Messick’s framework [54–56]. The items in these assessment tools could be the inspiration for the items in a curriculum. In the seven studies using an assessment tool all of them were checklists and the validity evidence was not explored for any of these checklists [22, 28, 30, 31, 47, 52, 53].

### Educational methods

A key element of curriculum development is addressing different educational strategies or adding educational components to improve the learning process. The mastery learning approach builds on the concept that not time but acquired competencies define the educational process [84]. Usually, by introducing new steps of procedures or topics when a previous step has been mastered. This could be introduction, machine settings and then ultrasound scanning a forearm followed by training cannulation technique on phantoms. The approach can differ and some suggest an effect by letting participants self-evaluate when they are ready to move on based on fixed goals [85, 86]. Others prefer letting an external objective evaluation and feedback by a simulator or instructor decide when to move on [87]. Feedback as a mechanism is a central part of the mastery-learning concept with its possibilities to improve performance by correction. On the other hand, too much feedback might also have its drawbacks as explained by the guidance hypotheses [84, 88, 89].

Mastery learning has proven efficacy but surprisingly only three studies used this approach [28, 30, 31]. A reason could be that it is easier to plan a traditional course using a fixed amount of time than a course that ensures that all trainees acquire the pre-defined proficiency level because trainees learn at different paces. The three studies prove that it is possible to plan an USG-PVC education by mastery learning. Compared to the studies that did not use mastery learning, mastery learning did not take up more time, with an average around 1 h, with approximate 30 min extra education time, if the first assessment was not passed [28, 30, 31]. Additionally, Kule et al. showed that so called overtraining did not increase success rate [31].

Four studies address the learning curve of nurses after an education program [41–43, 52]. Three studies showed a steep learning curve but a difference in attempts to obtain a success rate of 70–80% between four and ten USG-PVC performed in the clinic [41, 52, 73]. This finding aligns with the superiority of the mastery learning approach; no fixed numbers can ensure competency of all trainees.

Evaluation of different educational strategies and educational initiatives is equally important as investigating the effect of education. Chenkin et al. found that a one-hour web-based learning program was equally efficient compared to a one-hour traditional classroom lecture [38]. Only three of the studies used an e-learning module as a part of their educational program [22, 36, 47]. This indicates that e-learning is not implemented to its capability. The usage of color Doppler function to identify “twinkle artifacts” had an effect on cannulation time and a positive but non-significant impact on the success rate [40]. New studies in the future can help to clarify the full effect of color Doppler in USG-PVC education.

Neither test-enhanced learning nor using different types of phantoms seem to have a significant effect on the learning outcome and therefor none of these can be recommended [37, 39, 50].

### Clinical impact

The program’s clinical impact is the highest level of evaluation in Kirkpatrick’s method for evaluating the effects of educational programs [90]. The measurable impact of an educational program on an institutional level defines the impact of the program and evaluates the potential benefits and cost-efficiency.

The majority of the included studies found that ultrasound-guidance improved PVC-placement in either attempts, time, or success rate compared to traditional method when used on DIVA patients [19, 32, 52, 60–63] as it is also mentioned in Van-Loon meta-analysis [91]. Furthermore, a decrease in the need for CVC or PICC was seen in several studies [21, 29, 62, 66, 67]. Carter et al. and Oliveira et al. found that the effectiveness did not depend on profession and education should therefore be considered to a wide range of health care professions [25, 68].

Even though most studies found an effect on success rate, time, or amount of attempts, the results varied a lot between studies and few did not find an effect. The variation of success rate was between 85 and 97.5% in the top group [17, 19, 22, 24, 42, 53, 59, 64, 68], down to only 63% in adult patients [58] and no difference between traditional and ultrasound-guided in two studies [65, 69]. These big differences seem strange and even though some are explained by difference in equipment, patients and practitioners, some of the differences might also be explained by different educational strategies and the lack of evidence-based curricula to ensure competencies in ultrasound-guided PVC-placement. This shows the importance of more studies and easy access to information about proper educational strategies for USG-PVC.

### Limitations

This systematic review was conducted on the premises of the available published articles and their quantity and quality. Only including full text published articles could have resulted in the exclusion of possibly relevant results. Ideally, a meta-analysis of presented data should have been included as a part of this review, but was deemed not clinically meaningful due to the large heterogeneity of the reported methods and results.

### Implications

In summary there seems to be a clinical effect of using USG-PIV in specific patient groups as DIVA, and maybe also children. An educational program could very well be structured around the mastery-learning program, with an e-learning pre-course introducing the participants to the principals of ultrasound, anatomy and USG-PVC technique. This e-learning could be followed by a hands-on session structured around mastery learning and ending with an assessment test, at the moment preferably the one from Primdahl et al. since it is the only one where validity of evidence has been explored.

## Conclusion

Current studies suggest a potential benefit of ultrasound guided USG-PVC training on success rate, procedure time, cannulation attempts, and by reducing the need for subsequent CVC or PICC in adult patients. An assessment tool with proven validity of evidence to ensure competence exists and educational strategies like mastery learning, e-learning and the usage of color Doppler show promising results but an evidence-based USG-PVC-placement training program using these strategies combined is still warranted.

## Supplementary Information

**Additional file 1.** General search strategy.

**Additional file 2.** Full search strategy.

**Additional file 3. **Data extraxtion.

## Data Availability

All extracted data not included in the article text is available in the additional file.

## References

[1] Fields JM, Piela NE, Au AK, Ku BS (2014). Risk factors associated with difficult venous access in adult ED patients. Am J Emerg Med.

[2] Pinto A, Pinto F, Faggian A (2013). Sources of error in emergency ultrasonography. Crit Ultrasound J.

[3] McMillan HJ, Writer H, Moreau KA (2016). Lumbar puncture simulation in pediatric residency training: improving procedural competence and decreasing anxiety. BMC Med Educ.

[4] Evans LV, Dodge KL, Shah TD, Kaplan LJ, Siegel MD, Moore CL, Hamann CJ, Lin Z, DʼOnofrio G (2010). Simulation training in central venous catheter insertion: improved performance in clinical practice. Acad Med.

[5] Madenci AL, Solis CV, de Moya MA (2014). Central venous access by trainees: a systematic review and meta-analysis of the use of simulation to improve success rate on patients. Simul Healthc.

[6] Pietersen PI, Madsen KR, Graumann O, Konge L, Nielsen BU, Laursen CB (2018). Lung ultrasound training: a systematic review of published literature in clinical lung ultrasound training. Crit Ultrasound J.

[7] Kahr Rasmussen N, Andersen TT, Carlsen J, et al. Simulation-based training of ultrasound-guided procedures in radiology - a systematic review. Ultraschall Med. 2019;40(5):584–602. 10.1055/a-0896-2714. 10.1055/a-0896-2714 31083742

[8] Moher D, Liberati A, Tetzlaff J, Altman DG, PRISMA Group (2009). Preferred reporting items for systematic reviews and meta-analyses: the PRISMA statement. J Clin Epidemiol.

[9] Higgins JPT, Altman DG, Gøtzsche PC (2011). The Cochrane Collaboration’s tool for assessing risk of bias in randomised trials. BMJ..

[10] GA Wells, Shea, B, D O’Connell, J Peterson, V Welch, M Losos, P Tugwell. The Newcastle-Ottawa Scale (NOS) for assessing the quality of nonrandomised studies in meta-analyses [Available from: http://www.ohri.ca/programs/clinical_epidemiology/oxford.asp]. Accessed 13 Mar 2018.

[11] Osborn SR, Borhart J, Antonis MS (2012). Medical students benefit from the use of ultrasound when learning peripheral IV techniques. Crit Ultrasound J.

[12] Edwards C, Jones J (2018). Development and implementation of an ultrasound-guided peripheral intravenous catheter program for emergency nurses. J Emerg Nurs.

[13] Ng C, Ng L, Kessler DO (2017). Attitudes towards three ultrasound-guided vascular access techniques in a paediatric emergency department. Br J Nurs.

[14] Adhikari S, Schmier C, Marx J (2015). Focused simulation training: emergency department nurses’ confidence and comfort level in performing ultrasound-guided vascular access. J Vasc Access.

[15] Breslin R, Collins K, Cupitt J (2018). The use of ultrasound as an adjunct to peripheral venous cannulation by junior doctors in clinical practice. Med Teach.

[16] Costantino TG, Kirtz JF, Satz WA (2010). Ultrasound-guided peripheral venous access vs. the external jugular vein as the initial approach to the patient with difficult vascular access. J Emerg Med.

[17] Salleras-Duran L, Fuentes-Pumarola C, Bosch-Borras N (2016). Ultrasound-guided peripheral venous catheterization in emergency services. J Emerg Nurs.

[18] Panebianco NL, Fredette JM, Szyld D, Sagalyn EB, Pines JM, Dean AJ (2009). What you see (sonographically) is what you get: vein and patient characteristics associated with successful ultrasound-guided peripheral intravenous placement in patients with difficult access. Acad Emerg Med.

[19] Costantino TG, Parikh AK, Satz WA, Fojtik JP (2005). Ultrasonography-guided peripheral intravenous access versus traditional approaches in patients with difficult intravenous access. Ann Emerg Med.

[20] Desai K, Vinograd AM, Abbadessa MKF, Chen AE (2018). Longevity and complication rates of ultrasound guided versus traditional peripheral intravenous catheters in a pediatric emergency department. J Assoc Vasc Access.

[21] Shokoohi H, Boniface K, McCarthy M, Khedir al-tiae T, Sattarian M, Ding R, Liu YT, Pourmand A, Schoenfeld E, Scott J, Shesser R, Yadav K (2013). Ultrasound-guided peripheral intravenous access program is associated with a marked reduction in central venous catheter use in noncritically ill emergency department patients. Ann Emerg Med.

[22] Duran-Gehring P, Bryant L, Reynolds JA, Aldridge P, Kalynych CJ, Guirgis FW (2016). Ultrasound-guided peripheral intravenous catheter training results in physician-level success for emergency department technicians. J Ultrasound Med.

[23] Durand-Bailloud L, Aho LS, Savoldelli G, Ecarnot F, Girard C, Benkhadra M (2017). Non-dominant hand quicker to insert peripheral venous catheters under echographic guidance: a randomised trial. Anaesth, Crit Care Pain Med.

[24] Brannam L, Blaivas M, Lyon M, Flake M (2004). Emergency nurses’ utilization of ultrasound guidance for placement of peripheral intravenous lines in difficult-access patients. Acad Emerg Med Off J Soc Acad Emerg Med.

[25] Oliveira L, Lawrence M (2016). Ultrasound-guided peripheral intravenous access program for emergency physicians, nurses, and corpsmen (technicians) at a military hospital. Mil Med.

[26] Blaivas M, Lyon M (2006). The effect of ultrasound guidance on the perceived difficulty of emergency nurse-obtained peripheral IV access. J Emerg Med.

[27] Blick C, Vinograd A, Chung J, Nguyen E, Abbadessa MKF, Gaines S, Chen A (2021). Procedural competency for ultrasound-guided peripheral intravenous catheter insertion for nurses in a pediatric emergency department. J Vasc Access.

[28] Ballard HA, Tsao M, Robles A, et al. Use of a simulation-based mastery learning curriculum to improve ultrasound-guided vascular access skills of pediatric anesthesiologists. Paediatr Anaesth. 2020;30(11):1204–10. 10.1111/pan.13953. 10.1111/pan.13953 32594590

[29] Galen B, Baron S, Young S, Hall A, Berger-Spivack L, Southern W (2020). Reducing peripherally inserted central catheters and midline catheters by training nurses in ultrasound-guided peripheral intravenous catheter placement. BMJ Qual Saf.

[30] Amick AE, Feinsmith SE, Davis EM, et al. Simulation-Based Mastery Learning Improves Ultrasound-Guided Peripheral Intravenous Catheter Insertion Skills of Practicing Nurses. Simul Healthc. 2021; Publish Ahead of Print. 10.1097/SIH.0000000000000545 33428356

[31] Kule A, Richards RA, Vazquez HM, et al. Medical Student Ultrasound-Guided Intravenous Catheter Education: A Randomized Controlled Trial of Overtraining in a Simulation-Based Mastery Learning Setting. Simul Healthc. 2021. 10.1097/SIH.0000000000000554. Epub ahead of print. 10.1097/SIH.0000000000000554 33534403

[32] Bahl A, Pandurangadu AV, Tucker J, Bagan M (2016). A randomized controlled trial assessing the use of ultrasound for nurse-performed IV placement in difficult access ED patients. Am J Emerg Med.

[33] Batten S, Pazdernik V, Schneider R, Kondrashova T (2020). Interprofessional approach to learning vascular access with ultrasonography by medical students and nurses. Mo Med.

[34] Clemmesen L, Knudsen L, Sloth E (2012). Dynamic needle tip positioning - ultrasound guidance for peripheral vascular access. A randomized, controlled and blinded study in phantoms performed by ultrasound novices. Ultraschall Med.

[35] Tassone HM, Tayal VS, Weekes AJ (2012). Ultrasound-guided oblique approach for peripheral venous access in a phantom model. Crit Ultrasound J.

[36] Reeves T, Morrison D, Altmiller G (2017). A nurse-led ultrasound-enhanced vascular access preservation program. Am J Nurs.

[37] Slomer A, Chenkin J (2017). Does test-enhanced learning improve success rates of ultrasound-guided peripheral intravenous insertion? A randomized controlled trial. AEM Educ Train.

[38] Chenkin J, Lee S, Huynh T, Bandiera G (2008). Procedures can be learned on the web: a randomized study of ultrasound-guided vascular access training. Acad Emerg Med Off J Soc Acad Emerg Med.

[39] Davis J, Faust T, Tajani A, Bates A, Jarriel J, Au A, Fields JM (2017). A randomized study of training with large versus small vessel size on successful ultrasound-guided peripheral venous access. J Vasc Access.

[40] Gardecki J, Hughes LP, Zakaria S, et al. Use of the color Doppler twinkle artifact for teaching ultrasound guided peripheral vascular access. J Vasc Access. 2020;22:1129729820959907. 10.1177/1129729820959907. Epub ahead of print. 10.1177/1129729820959907 32962536

[41] Anderson AP, Taroc AM, Wang X, Beardsley E, Solari P, Klein EJ. Ultrasound guided peripheral IV placement: an observational study of the learning curve in pediatric patients. J Vasc Access. 2021:112972982098795. 10.1177/1129729820987958. 10.1177/1129729820987958 33467970

[42] Stolz LA, Cappa AR, Minckler MR, Stolz U, Wyatt RG, Binger CW, Amini R, Adhikari S (2016). Prospective evaluation of the learning curve for ultrasound-guided peripheral intravenous catheter placement. J Vasc Access.

[43] Ault MJ, Tanabe R, Rosen BT (2015). Peripheral intravenous access using ultrasound guidance: defining the learning curve. J Assoc Vasc Access.

[44] Griffiths J, Carnegie A, Kendall R, Madan R (2017). A randomised crossover study to compare the cross-sectional and longitudinal approaches to ultrasound-guided peripheral venepuncture in a model. Crit Ultrasound J.

[45] Erickson CS, Liao MM, Haukoos JS, Douglass E, DiGeronimo M, Christensen E, Hopkins E, Bender B, Kendall J (2014). Ultrasound-guided small vessel cannulation: long-axis approach is equivalent to short-axis in novice sonographers experienced with landmark-based cannulation. West J Emerg Med.

[46] Stone MB, Moon C, Sutijono D, Blaivas M (2010). Needle tip visualization during ultrasound-guided vascular access: short-axis vs long-axis approach. Am J Emerg Med.

[47] Good RJ, Rothman KK, Ackil DJ, Kim JS, Orsborn J, Kendall JL (2019). Hand motion analysis for assessment of nursing competence in ultrasound-guided peripheral intravenous catheter placement. J Vasc Access.

[48] Thorn S, Aagaard Hansen M, Sloth E, Knudsen L (2017). Guidance markers increase the accuracy of simulated ultrasound-guided vascular access: an observational cohort study in a phantom. J Vasc Access.

[49] Fürst RV, Polimanti AC, Galego SJ (2017). Ultrasound-guided vascular access simulator for medical training: proposal of a simple, economic and effective model. World J Surg.

[50] Leung KY, Mok KL, Yuen CK, Wong YT, Kan PG (2016). Evaluation of a commercial phantom and pork meat model for simulation based training of ultrasound guided intravenous catheterisation. Hong Kong J Emerg Med.

[51] Vitto MJ, Myers M, Vitto CM, Evans DP (2016). Perceived difficulty and success rate of standard versus ultrasound-guided peripheral intravenous cannulation in a novice study group: a randomized crossover trial. J Ultrasound Med.

[52] Feinsmith S, Huebinger R, Pitts M (2018). Outcomes of a simplified ultrasound-guided intravenous training course for emergency nurses. J Emerg Nurs.

[53] Moore C (2013). An emergency department nurse-driven ultrasound-guided peripheral intravenous line program. J Assoc Vasc Access.

[54] Primdahl SC, Todsen T, Clemmesen L, Knudsen L, Weile J (2016). Rating scale for the assessment of competence in ultrasound-guided peripheral vascular access - a Delphi consensus study. J Vasc Access.

[55] Jung CF, Breaud AH, Sheng AY, Byrne MW, Muruganandan KM, Dhanani M, Leo MM (2016). Delphi method validation of a procedural performance checklist for insertion of an ultrasound-guided peripheral intravenous catheter. Am J Emerg Med.

[56] Primdahl SC, Weile J, Clemmesen L, Madsen KR, Subhi Y, Petersen P, Graumann O (2018). Validation of the peripheral ultrasound-guided vascular access rating scale. Medicine..

[57] Blaivas M, Brannam L, Fernandez E (2003). Short-axis versus long-axis approaches for teaching ultrasound-guided vascular access on a new inanimate model. Acad Emerg Med Off J Soc Acad Emerg Med.

[58] Chinnock B, Thornton S, Hendey GW (2007). Predictors of success in nurse-performed ultrasound-guided cannulation. J Emerg Med.

[59] Mahler SA, Wang H, Lester C, Skinner J, Arnold TC, Conrad SA (2011). Short- vs long-axis approach to ultrasound-guided peripheral intravenous access: a prospective randomized study. Am J Emerg Med.

[60] Oakley E, Wong AM (2010). Ultrasound-assisted peripheral vascular access in a paediatric ED. Emerg Med Aust.

[61] Bauman M, Braude D, Crandall C (2009). Ultrasound-guidance vs. standard technique in difficult vascular access patients by ED technicians. Am J Emerg Med.

[62] Partovi-Deilami K, Nielsen JK, Moller AM, Nesheim SS, Jorgensen VL (2016). Effect of ultrasound-guided placement of difficult-to-place peripheral venous catheters: a prospective study of a training program for nurse anesthetists. AANA J.

[63] Doniger SJ, Ishimine P, Fox JC, Kanegaye JT (2009). Randomized controlled trial of ultrasound-guided peripheral intravenous catheter placement versus traditional techniques in difficult-access pediatric patients. Pediatr Emerg Care.

[64] Sou V, McManus C, Mifflin N, Frost SA, Ale J, Alexandrou E (2017). A clinical pathway for the management of difficult venous access. BMC Nurs.

[65] Bridey C, Thilly N, Lefevre T, Maire-Richard A, Morel M, Levy B, Girerd N, Kimmoun A (2018). Ultrasound-guided versus landmark approach for peripheral intravenous access by critical care nurses: a randomised controlled study. BMJ Open.

[66] Maiocco G, Coole C (2012). Use of ultrasound guidance for peripheral intravenous placement in difficult-to-access patients: advancing practice with evidence. J Nurs Care Qual.

[67] Gopalasingam N, Thomsen AE, Folkersen L (2017). A successful model to learn and implement ultrasound-guided venous catheterization in apheresis. J Clin Apher.

[68] Carter T, Conrad C, Wilson JL (2015). Ultrasound guided intravenous access by nursing versus resident staff in a community based teaching hospital: a “noninferiority” trial. Emerg Med Int.

[69] Bair AE, Rose JS, Vance CW, Andrada-Brown E, Kuppermann N (2008). Ultrasound-assisted peripheral venous access in young children: a randomized controlled trial and pilot feasibility study. West J Emerg Med.

[70] Schoenfeld E, Shokoohi H, Boniface K (2011). Ultrasound-guided peripheral intravenous access in the emergency department: patient-centered survey. West J Emerg Med.

[71] Vinograd AM, Zorc JJ, Dean AJ, Abbadessa MKF, Chen AE (2018). First-attempt success, longevity, and complication rates of ultrasound-guided peripheral intravenous catheters in children. Pediatr Emerg Care.

[72] Dargin JM, Rebholz CM, Lowenstein RA, Mitchell PM, Feldman JA (2010). Ultrasonography-guided peripheral intravenous catheter survival in ED patients with difficult access. Am J Emerg Med.

[73] Blick C, Vinograd A, Chung J, et al. Procedural competency for ultrasound-guided peripheral intravenous catheter insertion for nurses in a pediatric emergency department. J Vasc Access. 2021;22(2):232–7. 10.1177/1129729820937131. Epub 2020 Jun 27. 10.1177/1129729820937131 32597357

[74] Bortman J, Mahmood F, Mitchell J, Feng R, Baribeau Y, Wong V, Coolidge B, Bose R, Gao Z, Jones S, Matyal R (2019). Ultrasound-guided intravenous line placement course for certified registered nurse anesthetists: a necessary next step. AANA J.

[75] Kaganovskaya M, Wuerz L (2021). Development of an educational program using ultrasonography in vascular access for nurse practitioner students. Br J Nurs.

[76] Khamis NN, Satava RM, Alnassar SA, Kern DE (2016). A stepwise model for simulation-based curriculum development for clinical skills, a modification of the six-step approach. Surg Endosc.

[77] Bjerrum F, Thomsen ASS, Nayahangan LJ, Konge L (2018). Surgical simulation: current practices and future perspectives for technical skills training. Med Teach.

[78] Nayahangan LJ, Stefanidis D, Kern DE, Konge L (2018). How to identify and prioritize procedures suitable for simulation-based training: experiences from general needs assessments using a modified Delphi method and a needs assessment formula. Med Teach.

[79] Bessmann EL, Ostergaard HT, Nielsen BU (2019). Consensus on technical procedures for simulation-based training in anaesthesiology: a Delphi-based general needs assessment. Acta Anaesthesiol Scand.

[80] Ahern M, Mallin MP, Weitzel S, Madsen T, Hunt P (2010). Variability in ultrasound education among emergency medicine residencies. West J Emerg Med.

[81] Clayton MJ (1997). Delphi: a technique to harness expert opinion for critical decision-making tasks in education. Educ Psychol.

[82] Messick S (1995). Validity of psychological assessment: validation of inferences from persons’ responses and performances as sceintific inquiry into score meaning. Am Psychol.

[83] Downing SM. Validity: On the meaningful interpretation of assessment data. Med Educ. 2003;37(9):830–7. 10.1046/j.1365-2923.2003.01594.x. 10.1046/j.1365-2923.2003.01594.x 14506816

[84] Cook DA, Brydges R, Zendejas B, Hamstra SJ, Hatala R (2013). Mastery learning for health professionals using technology-enhanced simulation: a systematic review and meta-analysis. Acad Med.

[85] Brydges R, Butler D. A reflective analysis of medical education research on self-regulation in learning and practice. Med Educ. 2012;46(1):71–9. 10.1111/j.1365-2923.2011.04100.x. 10.1111/j.1365-2923.2011.04100.x 22150198

[86] Brydges R, Carnahan H, Rose D, et al. Comparing self-guided learning and educator-guided learning formats for simulation-based clinical training. Journal of advanced nursing. 2010;66(8):1832–44. 10.1111/j.1365-2648.2010.05338.x. 10.1111/j.1365-2648.2010.05338.x 20557388

[87] Snyder CW, Vandromme MJ, Tyra SL, et al. Effects of virtual reality simulator training method and observational learning on surgical performance. World J Surg. 2011;35(2):245–52. 10.1007/s00268-010-0861-1. 10.1007/s00268-010-0861-1 21086125

[88] Lee TD, White MA, Carnahan H (1990). On the role of knowledge of results in motor learning. J Mot Behav.

[89] Stefanidis D, Korndorffer JR, Heniford BT, Scott DJ (2007). Limited feedback and video tutorials optimize learning and resource utilization during laparoscopic simulator training. Surgery..

[90] Kirkpatrick D, Kirkpatrick J. Evaluating training programs: the four levels. Oakland: Berrett-Koehler Publishers; 2006.

[91] van Loon FHJ, Buise MP, Claassen JJF, Dierick-van Daele ATM, Bouwman ARA (2018). Comparison of ultrasound guidance with palpation and direct visualisation for peripheral vein cannulation in adult patients: a systematic review and meta-analysis. Br J Anaesth.

