# Supplementary material for: Education in the placement of ultrasound-guided peripheral venous catheters: a systematic review
Source: Scand J Trauma Resusc Emerg Med. 2021 Jun 27;29:83. doi: 10.1186/s13049-021-00897-z (PMC8237454; doi:10.1186/s13049-021-00897-z)
Supplement: Supplementary file 1 — Additional file 1. General search strategy. [file 13049_2021_897_MOESM1_ESM.docx]

**Additional file** 1: General search strategy

((("peripheral venous catheter" OR PVC OR "intravenous catheter" OR I.V. OR IV OR "venous access" OR "vein catheterization" OR "Vein access")) AND ("ultrasound guided" OR sonograph* OR ultrasound OR ultrasonic OR ultrasound-guided OR ultrasonograph* OR echograph*)) AND (Simulat* OR education OR training OR learn*)
