# Supplementary material for: Education in the placement of ultrasound-guided peripheral venous catheters: a systematic review
Source: Scand J Trauma Resusc Emerg Med. 2021 Jun 27;29:83. doi: 10.1186/s13049-021-00897-z (PMC8237454; doi:10.1186/s13049-021-00897-z)
Supplement: Supplementary file 2 — Additional file 2. Full search strategy. [file 13049_2021_897_MOESM2_ESM.docx]

**Additional file** 2: Full search strategy

**PubMed:**
(("peripheral venous catheter"[All Fields] OR PVC[All Fields] OR "intravenous catheter"[All Fields] OR I.V.[All Fields] OR IV[All Fields] OR "venous access"[All Fields] OR "vein catheterization"[All Fields] OR "Vein access"[All Fields]) AND ("ultrasound guided"[All Fields] OR (sonograph[All Fields] OR sonographed[All Fields] OR sonographer[All Fields] OR sonographer'[All Fields] OR sonographer's[All Fields] OR sonographers[All Fields] OR sonographers'[All Fields] OR sonographersf[All Fields] OR sonographi[All Fields] OR sonographia[All Fields] OR sonographias[All Fields] OR sonographic[All Fields] OR sonographica[All Fields] OR sonographicaally[All Fields] OR sonographical[All Fields] OR sonographicall[All Fields] OR sonographically[All Fields] OR sonographicaly[All Fields] OR sonographicelastographic[All Fields] OR sonographicevaluation[All Fields] OR sonographicfetal[All Fields] OR sonographich[All Fields] OR sonographicly[All Fields] OR sonographics[All Fields] OR sonographicum[All Fields] OR sonographie[All Fields] OR sonographieabteilung[All Fields] OR sonographieausbildung[All Fields] OR sonographiebefunde[All Fields] OR sonographiebefunden[All Fields] OR sonographiebefundes[All Fields] OR sonographiegerat[All Fields] OR sonographiegeraten[All Fields] OR sonographiegesteuerte[All Fields] OR sonographiegesteuerten[All Fields] OR sonographiegesteuertes[All Fields] OR sonographiekontrolle[All Fields] OR sonographiekurse[All Fields] OR sonographieren[All Fields] OR sonographiers[All Fields] OR sonographies[All Fields] OR sonographiescreening[All Fields] OR sonographiestudie[All Fields] OR sonographiesystem[All Fields] OR sonographiesystemen[All Fields] OR sonographifically[All Fields] OR sonographika[All Fields] OR sonographique[All Fields] OR sonographiques[All Fields] OR sonographisch[All Fields] OR sonographische[All Fields] OR sonographischem[All Fields] OR sonographischen[All Fields] OR sonographischer[All Fields] OR sonographisches[All Fields] OR sonographist[All Fields] OR sonographistes[All Fields] OR sonographists[All Fields] OR sonographs[All Fields] OR sonography[All Fields] OR sonography'[All Fields] OR sonography's[All Fields] OR sonographya[All Fields] OR sonographyally[All Fields] OR sonographyand[All Fields] OR sonographyc[All Fields] OR sonographycally[All Fields] OR sonographyers[All Fields] OR sonographyguided[All Fields] OR sonographyhas[All Fields] OR sonographyic[All Fields] OR sonographylaboratory[All Fields] OR sonographytrade[All Fields] OR sonographyy[All Fields]) OR ("diagnostic imaging"[Subheading] OR ("diagnostic"[All Fields] AND "imaging"[All Fields]) OR "diagnostic imaging"[All Fields] OR "ultrasound"[All Fields] OR "ultrasonography"[MeSH Terms] OR "ultrasonography"[All Fields] OR "ultrasound"[All Fields] OR "ultrasonics"[MeSH Terms] OR "ultrasonics"[All Fields]) OR ("ultrasonics"[MeSH Terms] OR "ultrasonics"[All Fields] OR "ultrasonic"[All Fields]) OR ultrasound-guided[All Fields] OR (ultrasonograph[All Fields] OR ultrasonograph'[All Fields] OR ultrasonographc[All Fields] OR ultrasonographer[All Fields] OR ultrasonographer'[All Fields] OR ultrasonographer's[All Fields] OR ultrasonographers[All Fields] OR ultrasonographguided[All Fields] OR ultrasonographi[All Fields] OR ultrasonographia[All Fields] OR ultrasonographias[All Fields] OR ultrasonographiaval[All Fields] OR ultrasonographic[All Fields] OR ultrasonographical[All Fields] OR ultrasonographically[All Fields] OR ultrasonographicaly[All Fields] OR ultrasonographics[All Fields] OR ultrasonographie[All Fields] OR ultrasonographied[All Fields] OR ultrasonographies[All Fields] OR ultrasonographique[All Fields] OR ultrasonographiques[All Fields] OR ultrasonographisch[All Fields] OR ultrasonographische[All Fields] OR ultrasonographischen[All Fields] OR ultrasonographischer[All Fields] OR ultrasonographisches[All Fields] OR ultrasonographist[All Fields] OR ultrasonographist's[All Fields] OR ultrasonographists[All Fields] OR ultrasonographly[All Fields] OR ultrasonographorin[All Fields] OR ultrasonographpy[All Fields] OR ultrasonographs[All Fields] OR ultrasonographv[All Fields] OR ultrasonography[All Fields] OR ultrasonography'[All Fields] OR ultrasonography's[All Fields] OR ultrasonography,[All Fields] OR ultrasonographyafter[All Fields] OR ultrasonographyalso[All Fields] OR ultrasonographyand[All Fields] OR ultrasonographyc[All Fields] OR ultrasonographycally[All Fields] OR ultrasonographyfusion[All Fields] OR ultrasonographyguided[All Fields] OR ultrasonographyic[All Fields] OR ultrasonographyically[All Fields] OR ultrasonographyimaging[All Fields] OR ultrasonographying[All Fields] OR ultrasonographynography[All Fields] OR ultrasonographys[All Fields] OR ultrasonographyto[All Fields] OR ultrasonographyzhejiang[All Fields]) OR (echograph[All Fields] OR echographe[All Fields] OR echographer[All Fields] OR echographers[All Fields] OR echographes[All Fields] OR echographi[All Fields] OR echographia[All Fields] OR echographiaval[All Fields] OR echographic[All Fields] OR echographical[All Fields] OR echographically[All Fields] OR echographicheskie[All Fields] OR echographics[All Fields] OR echographie[All Fields] OR echographie'[All Fields] OR echographied[All Fields] OR echographiees[All Fields] OR echographies[All Fields] OR echographique[All Fields] OR echographiquement[All Fields] OR echographiques[All Fields] OR echographisch[All Fields] OR echographische[All Fields] OR echographischen[All Fields] OR echographischer[All Fields] OR echographisches[All Fields] OR echographist[All Fields] OR echographist's[All Fields] OR echographiste[All Fields] OR echographistes[All Fields] OR echographists[All Fields] OR echographiwue[All Fields] OR echographs[All Fields] OR echography[All Fields] OR echography's[All Fields] OR echographyallow[All Fields] OR echographyc[All Fields] OR echographycally[All Fields]))) AND ((simulat[All Fields] OR simulata[All Fields] OR simulatability[All Fields] OR simulatable[All Fields] OR simulatae[All Fields] OR simulataeous[All Fields] OR simulatanagnosia[All Fields] OR simulataneous[All Fields] OR simulataneously[All Fields] OR simulatanously[All Fields] OR simulatanprophylaxe[All Fields] OR simulatated[All Fields] OR simulatd[All Fields] OR simulate[All Fields] OR simulate'[All Fields] OR simulatea[All Fields] OR simulateable[All Fields] OR simulated[All Fields] OR simulated'[All Fields] OR simulated'for[All Fields] OR simulatedby[All Fields] OR simulatedgastric[All Fields] OR simulatedhole[All Fields] OR simulatedlongitudinal[All Fields] OR simulatedly[All Fields] OR simulatedresults[All Fields] OR simulatedtransmission[All Fields] OR simulateexposure[All Fields] OR simulatem[All Fields] OR simulatenous[All Fields] OR simulatenously[All Fields] OR simulateon[All Fields] OR simulateous[All Fields] OR simulateously[All Fields] OR simulater[All Fields] OR simulates[All Fields] OR simulateted[All Fields] OR simulatethe[All Fields] OR simulateur[All Fields] OR simulateurs[All Fields] OR simulati[All Fields] OR simulatie[All Fields] OR simulatiemodel[All Fields] OR simulatieonderwijs[All Fields] OR simulaties[All Fields] OR simulatiespel[All Fields] OR simulatiib[All Fields] OR simulatilis[All Fields] OR simulatin[All Fields] OR simulatineous[All Fields] OR simulating[All Fields] OR simulating'[All Fields] OR simulatingcochlearimplants[All Fields] OR simulatingqymphosarcoma[All Fields] OR simulatingthe[All Fields] OR simulatio[All Fields] OR simulation[All Fields] OR simulation'[All Fields] OR simulation's[All Fields] OR simulation,[All Fields] OR simulation2008[All Fields] OR simulational[All Fields] OR simulationally[All Fields] OR simulationassociate[All Fields] OR simulationbased[All Fields] OR simulationcenter[All Fields] OR simulationed[All Fields] OR simulationen[All Fields] OR simulationg[All Fields] OR simulationgraphy[All Fields] OR simulationism[All Fields] OR simulationist[All Fields] OR simulationist'[All Fields] OR simulationists[All Fields] OR simulationjulich[All Fields] OR simulationof[All Fields] OR simulationplus[All Fields] OR simulationresults[All Fields] OR simulations[All Fields] OR simulations'[All Fields] OR simulationsa[All Fields] OR simulationsanalyse[All Fields] OR simulationsbaseret[All Fields] OR simulationsbasierte[All Fields] OR simulationsbasierten[All Fields] OR simulationsbasiertes[All Fields] OR simulationscenter[All Fields] OR simulationsdaniel[All Fields] OR simulationseinheiten[All Fields] OR simulationsergebnisse[All Fields] OR simulationsexperimente[All Fields] OR simulationsfilme[All Fields] OR simulationsfrage[All Fields] OR simulationsgrenzen[All Fields] OR simulationshilfe[All Fields] OR simulationskammer[All Fields] OR simulationsklinik[All Fields] OR simulationskonzept[All Fields] OR simulationslabor[All Fields] OR simulationsmaterialien[All Fields] OR simulationsmodell[All Fields] OR simulationsmodellen[All Fields] OR simulationsmodells[All Fields] OR simulationsmoglichkeiten[All Fields] OR simulationsmomodellen[All Fields] OR simulationsnachweis[All Fields] OR simulationsof[All Fields] OR simulationspatienten[All Fields] OR simulationsplus[All Fields] OR simulationsprobe[All Fields] OR simulationsproben[All Fields] OR simulationsprogramm[All Fields] OR simulationsprojekte[All Fields] OR simulationsprufung[All Fields] OR simulationsstudie[All Fields] OR simulationssystem[All Fields] OR simulationssysteme[All Fields] OR simulationssystemen[All Fields] OR simulationsszenarien[All Fields] OR simulationstechniken[All Fields] OR simulationsteknik[All Fields] OR simulationsthatthetwo[All Fields] OR simulationsthomas[All Fields] OR simulationstraening[All Fields] OR simulationstrainer[All Fields] OR simulationstraining[All Fields] OR simulationstrainings[All Fields] OR simulationstudies[All Fields] OR simulationsunterstutzten[All Fields] OR simulationsuntersuchung[All Fields] OR simulationsuntersuchungen[All Fields] OR simulationsverdacht[All Fields] OR simulationsverfahren[All Fields] OR simulationsversuch[All Fields] OR simulationsversuche[All Fields] OR simulationsversuchen[All Fields] OR simulationsvrsuch[All Fields] OR simulationswerkstatt[All Fields] OR simulationszentrum[All Fields] OR simulationszentrums[All Fields] OR simulationszentrumssowie[All Fields] OR simulationtemporal[All Fields] OR simulationto[All Fields] OR simulationtools[All Fields] OR simulationtrade[All Fields] OR simulationwhen[All Fields] OR simulatior[All Fields] OR simulative[All Fields] OR simulatively[All Fields] OR simulatn[All Fields] OR simulatnaously[All Fields] OR simulatnee[All Fields] OR simulatnees[All Fields] OR simulatneous[All Fields] OR simulatneously[All Fields] OR simulato[All Fields] OR simulatoins[All Fields] OR simulaton[All Fields] OR simulator[All Fields] OR simulator'[All Fields] OR simulator's[All Fields] OR simulator2[All Fields] OR simulator6[All Fields] OR simulatorbasierte[All Fields] OR simulatorbasierten[All Fields] OR simulatorbasiertes[All Fields] OR simulatorcentrum[All Fields] OR simulatore[All Fields] OR simulatorem[All Fields] OR simulatoren[All Fields] OR simulatorer[All Fields] OR simulatorgestutztes[All Fields] OR simulatori[All Fields] OR simulatormethode[All Fields] OR simulatormiljo[All Fields] OR simulatorn[All Fields] OR simulatoron[All Fields] OR simulatorplanung[All Fields] OR simulators[All Fields] OR simulators'[All Fields] OR simulatorstudie[All Fields] OR simulatortm[All Fields] OR simulatortrade[All Fields] OR simulatortraining[All Fields] OR simulatortraning[All Fields] OR simulatoru[All Fields] OR simulatorului[All Fields] OR simulatorunterstutzten[All Fields] OR simulatorversuch[All Fields] OR simulatory[All Fields] OR simulatray[All Fields] OR simulatrice[All Fields] OR simulatrix[All Fields] OR simulatsiia[All Fields] OR simulatsiiata[All Fields] OR simulatus[All Fields]) OR ("education"[Subheading] OR "education"[All Fields] OR "educational status"[MeSH Terms] OR ("educational"[All Fields] AND "status"[All Fields]) OR "educational status"[All Fields] OR "education"[All Fields] OR "education"[MeSH Terms]) OR ("education"[Subheading] OR "education"[All Fields] OR "training"[All Fields] OR "education"[MeSH Terms] OR "training"[All Fields]) OR (learn[All Fields] OR learn'[All Fields] OR learn123[All Fields] OR learn123's[All Fields] OR learn2cope[All Fields] OR learn2move[All Fields] OR learnability[All Fields] OR learnability'[All Fields] OR learnable[All Fields] OR learnable'[All Fields] OR learnals[All Fields] OR learnard[All Fields] OR learnatvivid[All Fields] OR learnbloodtransfusion[All Fields] OR learnbloodtransfusion'[All Fields] OR learnbyliving9[All Fields] OR learncoil[All Fields] OR learndent[All Fields] OR learndental[All Fields] OR learndirect[All Fields] OR learndsgns[All Fields] OR learne[All Fields] OR learned[All Fields] OR learned'[All Fields] OR learned''[All Fields] OR learnedness[All Fields] OR learnen[All Fields] OR learner[All Fields] OR learner'[All Fields] OR learner''[All Fields] OR learner's[All Fields] OR learner`s[All Fields] OR learneragent[All Fields] OR learnercentered[All Fields] OR learnercentred[All Fields] OR learnere[All Fields] OR learnerm[All Fields] OR learners[All Fields] OR learners'[All Fields] OR learners''most[All Fields] OR learners's[All Fields] OR learnerships[All Fields] OR learnerstrade[All Fields] OR learnes2[All Fields] OR learnet[All Fields] OR learney[All Fields] OR learnform[All Fields] OR learng[All Fields] OR learngaramkul[All Fields] OR learngdm[All Fields] OR learnharder[All Fields] OR learnhealth[All Fields] OR learnig[All Fields] OR learnign[All Fields] OR learnihan[All Fields] OR learnin[All Fields] OR learnin'[All Fields] OR learnine[All Fields] OR learning[All Fields] OR learning'[All Fields] OR learning''[All Fields] OR learning','inspired[All Fields] OR learning'h[All Fields] OR learning's[All Fields] OR learning1[All Fields] OR learning4excellence[All Fields] OR learninga[All Fields] OR learningand[All Fields] OR learningbased[All Fields] OR learningbonn[All Fields] OR learningcurve[All Fields] OR learningdesignsinc[All Fields] OR learningdl[All Fields] OR learningfirst[All Fields] OR learningfrom[All Fields] OR learninghandbook[All Fields] OR learningin[All Fields] OR learninglab[All Fields] OR learninglaboratory[All Fields] OR learninglinkoping[All Fields] OR learninglondonuk[All Fields] OR learningman369[All Fields] OR learningmemory[All Fields] OR learningpartnershipsonline[All Fields] OR learningplus[All Fields] OR learningprocess[All Fields] OR learningradiology[All Fields] OR learningrx[All Fields] OR learnings[All Fields] OR learningservices[All Fields] OR learningship[All Fields] OR learningteaching[All Fields] OR learningtm[All Fields] OR learningtrade[All Fields] OR learningu[All Fields] OR learninig[All Fields] OR learninq[All Fields] OR learnlab[All Fields] OR learnlab's[All Fields] OR learnlink[All Fields] OR learnmore[All Fields] OR learnmore'[All Fields] OR learnmtl[All Fields] OR learnning[All Fields] OR learnovation[All Fields] OR learnpaediatrics[All Fields] OR learnreduce[All Fields] OR learnring[All Fields] OR learns[All Fields] OR learns'[All Fields] OR learnsense[All Fields] OR learnsmart[All Fields] OR learnsomething[All Fields] OR learnsthe[All Fields] OR learnt[All Fields] OR learnt'[All Fields] OR learntand[All Fields] OR learntb[All Fields] OR learntibulo[All Fields] OR learntolive[All Fields] OR learntomove[All Fields] OR learntrade[All Fields] OR learnworld[All Fields] OR learny[All Fields] OR learnzone[All Fields]))

**Embase:**((((Peripheral venous catheter/) or (PVC.mp. [mp=title, abstract, heading word, drug trade name, original title, device manufacturer, drug manufacturer, device trade name, keyword, floating subheading word, candidate term word])or(Intravenous catheter.mp. [mp=title, abstract, heading word, drug trade name, original title, device manufacturer, drug manufacturer, device trade name, keyword, floating subheading word, candidate term word])or(IV.mp. [mp=title, abstract, heading word, drug trade name, original title, device manufacturer, drug manufacturer, device trade name, keyword, floating subheading word, candidate term word])or(Venous access.mp. [mp=title, abstract, heading word, drug trade name, original title, device manufacturer, drug manufacturer, device trade name, keyword, floating subheading word, candidate term word])or(vein catheterization/)or(Vein access.mp. [mp=title, abstract, heading word, drug trade name, original title, device manufacturer, drug manufacturer, device trade name, keyword, floating subheading word, candidate term word]))and((Ultrasound-guided.mp. [mp=title, abstract, heading word, drug trade name, original title, device manufacturer, drug manufacturer, device trade name, keyword, floating subheading word, candidate term word])or(ultrasound guided.mp. [mp=title, abstract, heading word, drug trade name, original title, device manufacturer, drug manufacturer, device trade name, keyword, floating subheading word, candidate term word])or(Sonograph*.mp. [mp=title, abstract, heading word, drug trade name, original title, device manufacturer, drug manufacturer, device trade name, keyword, floating subheading word, candidate term word])or(Echograph*.mp. [mp=title, abstract, heading word, drug trade name, original title, device manufacturer, drug manufacturer, device trade name, keyword, floating subheading word, candidate term word])or(Ultrasound/)or(ultrasonic.mp. [mp=title, abstract, heading word, drug trade name, original title, device manufacturer, drug manufacturer, device trade name, keyword, floating subheading word, candidate term word])or(ultrasonograph*.mp. [mp=title, abstract, heading word, drug trade name, original title, device manufacturer, drug manufacturer, device trade name, keyword, floating subheading word, candidate term word]))and((Simulat*.mp. [mp=title, abstract, heading word, drug trade name, original title, device manufacturer, drug manufacturer, device trade name, keyword, floating subheading word, candidate term word])or(education.mp. [mp=title, abstract, heading word, drug trade name, original title, device manufacturer, drug manufacturer, device trade name, keyword, floating subheading word, candidate term word])or(Training/)or(Learn*.mp. [mp=title, abstract, heading word, drug trade name, original title, device manufacturer, drug manufacturer, device trade name, keyword, floating subheading word, candidate term word])))Or((Peripheral venous catheter or PVC or Intravenous catheter or IV or Venous access or vein catheterization or Vein access) and (Ultrasound-guided or ultrasound guided or Sonograph* or Echograph* or Ultrasound or ultrasonic or ultrasonograph*) and (Simulat* or education or Training or Learn*)).mp. [mp=title, abstract, heading word, drug trade name, original title, device manufacturer, drug manufacturer, device trade name, keyword, floating subheading word, candidate term word))))

**Cinahl:**((Peripheral venous catheter or PVC or Intravenous catheter or IV or Venous access or vein catheterization or Vein access) and (Ultrasound-guided or ultrasound guided or Sonograph* or Echograph* or Ultrasound or ultrasonic or ultrasonograph*) and (Simulat* or education or Training or Learn*)).
