# Supplementary material for: Education in the placement of ultrasound-guided peripheral venous catheters: a systematic review
Source: Scand J Trauma Resusc Emerg Med. 2021 Jun 27;29:83. doi: 10.1186/s13049-021-00897-z (PMC8237454; doi:10.1186/s13049-021-00897-z)
Supplement: Supplementary file 3 — Additional file 3. Data extraxtion. [file 13049_2021_897_MOESM3_ESM.docx]

| **DATA-extraction for all included articles.** | | | | | | | | |
| --- | --- | --- | --- | --- | --- | --- | --- | --- |
| **Author** | **Design** | **Theoretical education** | | **Practical education** | **Assessment of competence*** | **Participants** | **Test objects** | **Clinical outcome** |
| Carter et al. (68) | RCT | Nurses: None | | Nurses: Hands-on followed by gel model training | None | **Nurses:** US novices, 11 nurses | **Patients:** adult DIVA | **Overall success rate:** 85.5%.  Nurses: 86% vs. residents 85%. Insignificant (p=0.305) |
|  |  | Residents: None | | Residents: On job training |  | **Doctor:** 5 residents |  |  |
| Tassone et al. (35) | RCT | None | | Five minutes "in service training" on gel-phantom model | None | **Doctor:** EM-attending or resident physicians | Commercial phantom | **First attempts success:** Transverse (T) = 70.8%, Longitudinal (L) = 83.3%, Oblique (O) = 45.8%.  Only significant difference between O and L (P=0.03).  **Time:** No significant difference **Attempts mean**: T = 1.75, L = 1.71 and O = 2.33 (Not significant) |
| Oakley et al. (60) | Observational study | Not clarified | | Training in basic principles of USG-PVC and practical experience using chicken-breast model. | None | **Doctor:** Emergency consultants | **Patients:**   children | Significant improvement in mean attempts, and success rate for difficult patients and patients under 10 years. |
| Duran-Gehring (22) | Cohort study | Instruction video (26 min.) and e-learning | | Training on live models and gel-phantoms | Post-test, 5 supervised PVC attempts | **ED-Technicians** | **Patients:**  adult DIVA | **Success rate;** 97.5%. 86.8% was placed in first attempt, 11.6% in second and 1.6% in third.  **Clinicians categorized**: 82.6% of patients to have DIVA and CVC avoided in 46.5% of the cases |
| Costantino et al. (16) | RCT | One month rotation in emergency ultrasound, including 16 hour didactic lectures and >150 total emergency ultrasound scans. (not specified length) | | | Not stated | **Doctor:** 2nd and 3nd year EM-residents | **Patients:**  adult DIVA | **Success rate:** US-guided 84% vs external jugular 50% (P=0.006). **Time:** No significant difference |
| Salleras-Duran et al. (17) | Not stated (observational) | Twenty-hour course covering basic concepts (eg. use of ultrasound equipment and echographic anatomy) Participants practiced the technique on simulators. | | | Not stated | **Nurse** | **Patients:**  adults | **Success rate:** 95.1% of patients, 84.2 % in first attempt. **Attempts** Mean: 1.18 |
| Panebianco et al. (18) | Prospective cohort study | Course including 16 hours of didactic lectures and over 150 technically adequate scans.  Residents had 4-week rotation in emergency US during their postgraduate year. | | | Ten supervised USG-PVC | **Doctor:** Residents | **Patients:**  adult DIVA | **Overall success rate:** 90%. First attempt success rate: 69%.  **Vessel Size:** Large size had a higher success rate.  **Vessel depth:** A depth under 1.6 cm has lower success rate.  Patient characteristics had no influence on success rate.  No difference between long-axis vs. short-axis approach. |
| Costantino et al. (19) | Cohort (described in study as RCT) | **Study training:** 1-hour didactic sessions of USG-PVC and CVC | | None | None | **Doctor:** Residents | **Patients:** adult DIVA | **Success rate:** USG-PVC 97% vs. traditional 33%.   **Time:** USG-PVC was Significantly faster. USG-PVC 4 min. vs. traditional 15 min.  Significantly fewer percutaneous punctures and higher patient satisfaction. |
|  |  | **Previous training**: 15 hour didactic lectures, 100 emergency ultrasonographic scans and a 3 week rotation in the ED | | |  |  |  |  |
| Bauman et al (61) | Cohort study | One hour didactic session on USG-PVC | | Hands on practice on gel-phantom | None | **ED-technicians**  >1 year experience | **Patients:** adult DIVA | **Success rate**: USG-PIV preformed significant better if the patients had more than 3 skin punctures prior to USG-PIV. **Time**: USG-PIV was twice as fast compared to traditional.  **Skin punctures:** Traditional method had two times the amount of skin punctures.  **Satisfaction:** 79 of 100 USG-PVC compared to 44 of 100 for traditional. |
| Blaivas et al. (57) | RCT | Thirty minutes didactic session on USG-PVC | | None | None | **Doctor:** EM-Residents, US novices | Home-made gel-phantom | **Mean time**: Short-axis took 2.36 minutes vs. long-axis that took 5.02 minutes (P=0.03). Skin penetrations, redirections or difficulty score showed no significant difference. |
| Griffiths et al. (44) | RCT | Thirty minutes didactic teaching session | | 5 min US scanning of upper extremities' vascular anatomy | None | **Medical student:** Senior students, with little to no prior experience. | Phantom | No significant difference in time, skin punctures or difficulty score between **short-axis** and **long-axis method**. |
| Durand-bailloud et al. (23) | RCT | Classroom setting, video and live demonstration | | None | None | **Nurse students** | Gel-phantom model | **Time:** Non-dominant hand for probe handling was significantly faster (p<0.001). 90% preferred dominant hand for probe handling. |
| Vitto et al. (51) | RCT | Traditional PVC:  Half-hour lecture.  USG-PVC:  Half-hour lecture. | | None | None | **Medical students:** Second-year | Standard peripheral PVC trainer | **Success rate:** 61/61 USG-PVC vs. 34/61 traditional PVC (P=0.001). USG-PIV preformed significant better in mean attempts (P<0.001) and difficulty score (P=0.003). |
|  |  |  |  |  |  |  | Standard USG-PVC trainer |  |
| Erickson et al. (44) | Observational study | One hour training | | Live-model training: Two practice cannulations on a phantom. | None | **Nurse**: 10 | Gel-phantom model | **Short-axis vs. long-axis Time** **(median):** No significant diff. **Success rate**: both 100% |
| Osborn et al. (11) | RCT | USG-PVC: 20 min. scripted tutorial | | None | None | **Medical student:** First- and second-year, US novices | Other students | **US vs. traditional approach**  **Attempts (mean):** No difference **Participants experience:** US significantly easier (P<0.01) |
|  |  | Traditional PVC: 20 min. scripted tutorial. | |  |  |  |  |  |
| Bair et al. (88) | RCT | **Physicians**: one-hour orientation of use of US-equipment | | **Physicians**: none | Each physician shows proficient technique on at least one adult and one child during training. | **Doctor:** Physician | **Patients:** adult DIVA and children | **Success rate:** ultrasound group 57% vs. 57% in traditional group. |
|  |  | **Nurses**: instructed in the procedure | | **Nurse**: none |  | **Nurse** |  |  |
| Stone et al. (46) | RCT | 20-min sessions on the proposed ultrasound-guided procedure | | None | None | **Medical students**: Senior | Gel-phantom model | **Needle tip visibility at time of vessel puncture:**  long-axis group 62% vs. short-axis 23% (p=0.01).  No significant difference in time to puncture. |
|  |  |  |  |  |  | **Doctor:** First-year emergency medicine residents |  |  |
| Chinnock et al. (58) | Observational study | 90-min training session, both one-person technique and two-person technique. | | | None | **Nurse** | **Patients:**  adult DIVA | **Overall cannulation success rate:** 63%.  No significant difference between one-person or two-person technique. |
| Moore et al (90) | Not addressed  (observational study) | **First 6 nurses:** 3 hour didactic and hands-on | | Afterwards spending 3 days with the peripheral IV team doing USG-PVC and helping in the ED. | Competency checklist. 25 success full cannulations. | **Nurse** | **Patients:** adult DIVA | From January to august, 2009, the percentage of success full cannulations for the original RN ranged from 88% to 100%.  During 2010 USG-PVC successfully placed was at least 90% and first attempt success was 81%. |
|  |  | **Other nurses**: 4 hour didactic and hands on | |  |  |  |  |  |
| Brannam et al (24) | Observational study | Lecture lasting 45 min., including still image and video. | | On an inanimate model simulating a deep peripheral arm vein. | None | **Nurse:** ED-nurse | **Patients:** not specified | **Overall success rate:** 87%  **Attempts (mean):** 2.2 pr. patient. (95% CI = 1.9 to 2.4) |
| Breslin et al. (15) | Cohort: Pre-post study | Didactic training and practical training, one week apart. 1) One-hour session of didactic teaching and supervised practice using phantoms. 2) One-hour session of supervised practice during which participants cannulated each other | | | Competence assessed using OSCE | **Doctor:** Foundation first-year doctors. | **Patients**:  Adults | **Success rate**: USG-PVC 73%.  **Cannulation attempts (mean)**: 1.8.  **Likert-scale:** Education components were; enjoyable 9.3, effective 9.6 and relevant 9.2. |
| Edwards et al (12) | Quality improvement project (observational) | 90 min of US-guidance and video demonstrations | | Unlimited hands-on training on gel-phantom model | Demonstrate competency | **Nurse** | **Patients:**  adult DIVA | 3 patients pr. shift with complicated vasculature.  A majority of nurses agreed that they now posses the abilities of USG-PVC and that the USG-PVC would help in their clinical work. |
| Schoenfeld et al. (91) | Observational study | Two hour training session: including a didactic presentation, scanning on live model arms and hands-on session on a gel-phantom model. | | | None | **ED-technicians** | **Patients:**  adult DIVA | **Patient satisfaction (mean):** Very happy, 9.2 points out of 10. USG-PVC compared to previous IV-methods, their happiness was 4.4 out of 5. |
| Oliveira et al (25) | Cohort study | 30 min. didactic session and online video material | | 90 min. hands-on session: live model, cannulation technique on a gelatine phantom | None | **Doctor**: 34 physicians | **Patients:**  adults | **Overall success rate**: Physicians 79.4%, nurses 63.2% corpsmen 50%. Corrected for experience levels no significant difference (P=0.13).  **Overall success:** long-axis approach 74.1%, short-axis approach 72% - combined approach 58,3%. Short-axis had the highest success rate for novice users. |
|  |  |  |  |  |  | **Nurse:** 19 emergency nurses |  |  |
|  |  |  |  |  |  | **ED-technicians:** 12 corpsmen |  |  |
| Clemmesen et al. (34) | RCT | One hour didactic session on USG-PVC with live demonstration. | | One hour hands-on workshop practicing on gel-phantoms. | None | Not stated other than: Little to no experience in USG-PVC | Gel-phantom  model | **Success rate:** dynamic needle tip positioning superior to long-axis (97% vs. 81%, p=0.01).  **Distance from centre:** dynamic needle tip positioning superior to long-axis (0.97 vs. 1.92 P= < 0.00001).  **Time:** long-axis was faster. |
| Ng. Carrie et al. (13) | Cross sectional study | One hour didactic training and instruction videos | | 90 min. practice session: cannulation on gel-phantom | None | **Nurse:** Paediatric ED-nurses | Gel-phantom model and patients | Just after training the one-person technique was the most preferred (41%), 3 month after 65% preferred the two-person self-guided technique. |
| Adhikari et al. (14) | Cross sectional study | Two hours simulation based training: didactic lectures, images, and videos. Practice sessions: human models and gel-phantom. | | | None | **Nurse:** ED-nurses, US novices. | None (questinary after training session) | All agreed that that it is possible for nurses to learn USG-PCV and it saves time, decreases discomfort, and increases patient satisfaction. |
| Partovi-deilami et al. (62) | Prospective cohort | One hour lecture | | One hour of training on live models and one hour USG-PVC on phantoms. | None | **Nurse:** 10 anaesthetic nurses | **Patients**: traditional PVC not possible for anaesthetic nurses. | **Success rate** rose from 0% to 83%.  **Reduction in:** time (p < 0.05), attempts (p < 0.05) and CVC placements from 11 out of 33 to 5 of 70. |
| Bahl et al; (32) | RCT | US-guidance: 1.5-hour didactic session with video. | | US-guidance: hands-on | 10 supervised USG-PVC placements | **Nurse**: ED nurses with at least 2 years experience | **Patients:** adult DIVA | **Success rate:** US-guided 76% and traditional 56% (P=0.02) No significant differences in time (p=0.75) or attempts (P=0.63). |
|  |  | Traditional method 1.5 hour didactic session | | Traditional method: none |  |  |  |  |
| Blaivas et al. (26) | Prospective observational study | 45 minutes lecture including still image and video | | Hands on practice: on inanimate model simulating a deep peripheral vein | None | **Nurse:** ED nurses with 1-22 years of experience. | Not stated (patients) | **Rating:** There was a statistically significant difference between the participants' perception of difficulty before and after US-guidance.  No significant difference between short-axis and long-axis method. |
| Desai et al. (20) | Cross-sectional study | Nurses and paediatric residents underwent a 4-hour course including a didactic component and hands on training. | | | None | **Nurses** and **Doctors:** paediatric residents | Patients | **Complication rates:** No significant difference between nurses and doctors. |
|  |  | EM residents, PEM fellows and PEM attending’s completed a rotation in emergency bedside ultrasound | | | None | **Doctors**: EM residents, PEM fellows and PEM attending’s |  |  |
| Vinograd et al. (73) | Prospective cohort study | Nurses and paediatric residents underwent a 4-hour course including a didactic component and hands on training. | | | None | **Nurses**  **Doctors:** Paediatric residents | **Patients:**  mainly children | **Success rate:** 68% first attempt, 87% second attempt and overall success rate was 91%. |
|  |  | EM-residents, fellows and attending’s placing USG-PVC all completed a prior rotation in emergency bedside ultrasound. | | |  | **Doctors:** EM residents, PEM fellows and PEM attending’s |  |  |
| Dargin et al. (90) | Observational study | One-hour lecture | | One-hour training session | Accredited in emergency ultrasound | **Doctors**: EM-residents or attending physicians | **Patients:**  adults | **Overall survival rate**: 56%  **Median survival time:** 26 hours  (76 patients). |
| Doniger et al. (63) | RCT | Physicians: one hour didactic training | | Physicians: 30 min individual workshop using a phantom | None | **Doctor:** physician | **Patients:** children | **Success rate:** USG-PIV vs. traditional no significant difference.  **Time (mean):** US-group 6.3 min vs. traditional 14.4min (p = <0.001)  **Attempts (median):** US group (1) vs. traditional (3)(P=0.004) |
|  |  | Nurses: 15 min didactic session and procedural workshop and practiced IV catheter placement on live patients. | | |  | **Nurse** |  |  |
| Mahler et al. (59) | RCT | Thirty min. lecture on USG-PVC | | One hour hands-on training on vascular phantoms | None | **Doctors:** 2 emergency attending physicians | **Patients:** adults | **Success rate:** short-axis 95% vs. long-axis 85%, no significant difference.  **Time (median):** 34 sec. short-axis group vs. 96 sec. long-axis group, (p =0.02). |
|  |  | The ED nurses all completed a course for USG-PVC | | | None | **Nurse: 2** ED-nurse |  |  |
| Thorn et al. (48) | Cohort study | Didactic session and hands-on training | | | None | **Nurses** | Phantom | Needle placement without the guidance markers: Physicians were better than nurses (p = 0.02). No significant differences when guidance markers were added (p = 0.80). All participants (n = 30) stated that the guidance markers were helpful. |
|  |  |  |  |  |  | **Doctors:** Physicians |  |  |
| Ault et al. (43) | Cohort study | Two-hour didactic session**,** 1:1 mentoring session. | | Two-hour 1:1 hands-on training on validated non-human tissue model | None | **Nurses** | **Patients:** included by physician's order | **Patient encounters required** for 10 successful PVC placements were 25 (18 to 30).  **Time (mean):** For cannulation19.57 min., ranging from 5 min. to 62 min.  Proficiency increased rapid during first 25 attempts and continued at a slower rate thereafter. |
| Stolz et al. (42) | Observational study | Two-hour session consisting of lecture-based didactics and hands-on training | | | None | **Nurses** | **Patients:** adult DIVA | **Success rate:** 88.24%,  15 attempts the success rate based on the final mixed effects logistic regression model is 88.2% |
|  |  |  |  |  |  | **Paramedics** |  |  |
| Slomer et al. (37) | RCT | Thirty min. didactic lecture and demonstration | | One-hour hands-on training. | After 10-14 days tested on phantom model | **Medical students** | Chicken-breast phantom | **Success rate:** Test enhanced group 93,3% (95% CI = 79.9-100%), vs. control group 80.0% (95% CI = 57,1%-100%) (p=0.6). |
|  |  |  |  | Test-enhanced: 45 min training, 15 min test. |  |  |  |  |
| Leung et al; (50) | Cohort study | Thirty min. didactic | | Thirty min. hands-on practice | None | **Doctors:** Physicians | Gel-phantom model and chicken-breast phantom | Median scores for all questions were identical except one; "feeling of cannulating a vessel resembling that of a human vessel" this showed that the chicken breast felt more resembled with humans vessel. |
| Davis et al (39) | RCT | One hour scripted presentation | | Hands on: randomized training on either a small vessel size phantom or big vessel size phantom. | None | **Medical students**: US novices | Large vessel phantom | No significant difference in: time to cannulation, success rate or attempts.  Data trended a favour for small vessel training (p = 0.15). |
| Feinsmith et al. (52) | Cohort study | An ER resident held a 4-hour didactic and hands-on course. | | | 10 successful USG-PVC attempts | **Nurses** | **Patients** | **Learning curve:** 1-10 attempts = success rate 81%, 11-20 attempts = success rate 84%, attempts 21-30 = success rate of 96%.  **Pre/post-training comparison:** decrease in DIVA attempts of 7% (P=0.003). |
| Miaocco et al. (66) | Not stated (Cohort study) | Two-hour course, didactic and hands-on | | | Not stated | **Nurses** | **Patients:**  not specified | **PICC (surgical):** decreased from 66 to 53% **First attempt success:** 71% |
| Shokoohi et al (21) | Prospective cohort study | Two-day general ultrasonography training, including didactics and hands-on practice and a two-week emergency-medicine ultrasonography rotation. | | | Mastery of technique | **Doctors:** Physicians | **Patients:** adult DIVA | During the 6-year period, the overall central venous catheter placement rate decreased by 80%, from 0.81% to 0.16%. The reduction was greater in non-critically ill patients. |
|  |  | ED technicians: two-hour training session, including live models arm and insertion on gel phantoms | | |  | **ED-technicians** |  |  |
| Fürst et al. (49) | Not clearly  stated (cross sectional) | Two-hour lecture | | Four-hour hands on training | None | **Medical students** | Chicken-breast phantom | Students had a general positive perception about every item asked. |
|  |  |  |  |  |  | **Doctors:** Freshmen graduate doctors |  |  |
| Sou et al. (64) | Cohort study | Didactic sessions held by CVC-service | | Hands-on training | Not clear | **Nurses** | **Patients:**  not specified | **Success rate:** First time success for cannulation was 93% (n = 348).  No more than 2 attempts required to gain venous access.  Significant difference in pain scores |
| Reeves et al. (36) | Cohort study | One-hour e-learning. Four-hours classroom education. | | Four-hours manufacturer provided instructional training. | 29 items checklist | **Nurses** | **Patients:**  infusion-patients | 15 nurse participants achieved a success rate of 85% or above, with a maximum of 2 attempts.  General decrease in attempts from 5.5 to 1.5. PICC reduced by 24% in medical-surgical unit and 8.4 % in the step-down unit. 40% reduction in non-essential PICC. |
| Primdahl et al. (56) | Prospective validity study | None | | None | Rated on the scale | **Doctors**: Physicians. Novices, intermediate and experts. | **Patients:**   not specified | Benchmark was set where the Novices and expert group overlapped at 29 points. |
| Chenkin et al.(38) | RCT | E-learning: one hour | | Two-hours independent practicing on live models and phantoms. | OSCE exam | **Doctors:** Junior EM-residents and EM-physicians, with introduction course in US. | **Commercial phantom:** OSCE based exam, pre-post questionaries’. | **OSCE score and Written exam**: no significant difference between e-learning and class-room.  A mean improvement for both groups in written (p < 0.001). |
|  |  | Class-room: one-hour class-room lecture covering the same material as the e-learning | |  |  |  |  |  |
| Gopalasingam et al. (67) | Prospective descriptive study | E-learning course in USG-PVC | | Practice on gel-phantom model and technique demonstration | 30 supervised catheterizations | **Nurses:** 6 specially trained apheresis nurses | **Patients**:  adults | 45,8% apheresis produced on CVCs pre-implementation. One year later 13,2% apheresis procedures were produced on CVCs. A significant decrease (P=0.001) Post-implementation no CVCs were placed due to failed PVC. |
| Bridey et al. (65) | RCT | One-hour lecture given by physician | | Practical placement | 4 supervised attempts | **Nurses** | **Patients**:  adults | **Success rate:** No difference between US and traditional method (p=0.618). |
| Good et al. (47) | Cohort study | E-learning | | Two-hour hands on | Tested by TSC (RICE) | **Nurses** | Phantom | Nurses improved in hand motion analysis after training, few nurses achieved expert level benchmarks for all 6 analysis. |
| Kaganovskaya et al. (92) | Quasiexperimental study (Pre-/post questionary) | One-hour didactic lecture. Topics: Anatomy, vein and artery selection on ultrasound | | Two-hour simulation course. Training ultrasound on a vein block (Phantom). | None | **Nurse students** | Pre-/post-test. Confidence survey | Pre-/post-test improvement from a mean score of 62% to a mean score of 78%.  86% of the students demonstrated an increase in self-confidence post course. |
| Batten et al. (33) | Prospective cohort (pre-/post-test study) | PowerPoint presentation explaining the relevance and objectives of ultrasound guided vein and a live demonstration. | | Cannulation technique training on commercial phantom. Upper extremity ultrasound scanning and supervised cannulation on other participants. | None | **Nurses** | Pre-/post-test  Confidence survey (Likert-scale) | **Pre-/post-test:** overall improvement of 45% **Confidence survey**: 50% felt capable of using a US-machine after the course, 67% of nurses felt capable of preforming an upper-extremity US-vascular examination and 67% of nurses found it difficult to interpret a 2D ultrasound image |
|  |  |  |  |  |  | **Medical students:** 2 years prior US education. |  | **Pre-/post-test:** overall improvement of 22% **Confidence survey**: 100% felt capable of using a US-machine after the course, 91% felt capable of preforming an upper extremity US-vascular examination, 9% found it difficult to interpret a 2D ultrasound image |
| Kule et al. (31) | Blinded RCT | 5 min introduction video | | One-on-one hands-on training on phantoms until passing score is met.  Then randomised in 3 groups. Each group overtraining 0, 4 or 8 extra passing attempts. | 19 item checklist | **Medical students** | Healthy volunteers | No significant difference between the 3 groups in the catheter placement or checklist performance. |
| Blick et al. (84) | Quality improvement project  (prospective cohort) | 30 min video, | | Two-four hour training session, training on both phantoms and live models. | 10 successful USG-PVC attempts | **Nurses** | Paediatric patients | First attempt success rate of 83%,  **Attempts for successful cannulation:** Median 1.1 attempt The probability of success immediately after training was 67% and increased to 83% by 10 unsupervised encounter. |
| Galen et al. (29) | Quality improvement project (Prospective cohort) | Video with following key components: Overview, Preparation, Placement of ultrasound-guided peripheral IV, and common errors | | Training with ultrasound device, both on live models and cannulation technique on commercial phantom.  All participants used less than 60 min. | 2 successful attempts on ultrasound mannequin. | **Nurses** | Patients | 97% of attempts were successful.  Pre-implementation: mean number of 4.8 PICC/midline catheters per month were placed. Under-Implementation: mean number of 2.5 PICC/midline catheters per month were placed. Post implementation: mean number of 4.3 PICC/midline catheters per month were placed. Comparison inpatient unit utilisation of PICC/midline catheters remained stable during all phases. |
| Amick et al. (30) | Prospective cohort | Step-by-step instruction video followed by recorded didactic lecture. | | The practice sessions consisted of structured exercises focusing on the basics of ultrasound, identification of vascular structures, needle tip control, and dynamic guidance | 30-items checklist | **Nurses** | Confidence survey  Pre-/post-test | 91,7% passed the checklist on first attempt.  Extra training time after a failed attempt between 15-60 minutes. Self-confidence: significant increase, mean of 2.32 before training to 3.85 after.  Success rate for cannulation: 89,5% Nurses with 0-2 years experience were more likely to complete the course |
| Anderson et al. (41) | Observational study | 30 min didactic session, presentation on inclusion criteria’s, identification of best veins, depth, diameter and proximity to nearby structures | | 90 min hands-on training on commercial phantoms.  Individualized feedback from instructor. | None | **Nurses** | Pediatric patients | Nine attempts to achieve 70% success rate for subsequent USG-PVC attempt.  Mixed effects logistic regression showed a positive correlation between number of attempts and success rate. |
| Bortman et al. (91) | Pre-/post-test cohort study | **Step 1**: 3 hour didactic online course, consisting of ultrasound physics, us equipment, knobology, vascular anatomy, ultrasound-guided vascular techniques,  **Step 2:** 2 days consisting of 3 hour training pr. day. Both days starting with a didactic session, followed by hands-on training including vessel characteristics, hand-eye coordination, in-plane and out-of-plane technique, both dynamic and static technique. Training on commercial phantom. | | | None | **Nurses:** Anaesthetists nurses | Post-test  Survey (likert-scale) | Pre- and post-test score increase from 59,13% to 70% (P=0.03) |
| Ballard et al. (28) | Pre-/post-test cohort study | Two-hour curriculum consisting of watching video on the indications, contraindications, and complications of ultrasound-guided intravenous catheter insertion and how to perform the procedure. | | Deliberate practice using live models (their peers) for ultrasound scanning and simulator for ultrasound-guided intravenous catheter insertion | 25 items checklist | **Doctors:** Paediatric anaesthesia physician | Pre-post self-confidence. | 16/76 met the checklist grade passing pre-intervention. 73/76 achieved the passing graded on first post-test. The 3 last meet the passing standard on a second post-test within 1 hour extra training.  Self-confidence levels improved from 3.2 to 3.9 (P<0.1). |
| Gardecki et al. (40) | Prospective randomized convenience study | 15 min didactic lecture, including ultrasound physics, machine operation, sono-anatomy, the process of USG-PVC placement in transverse view and depending of randomisation group the twinkle artefact | | 165 min Hands-on training with or without twinkle artefact. After training the participants was guided through two-successful ultrasound guided cannulations. | None | **Medicine student:** Pre-medicine students and medicine students | Commercial phantom | First pass success rate: No significant difference. TA: 82% Control: 57%  **Mean cannulation time:** Significant difference TA, 50.76 s (SD 26.93) versus control groups, 85.30 s (SD 65.47), p = 0.048. Posterior wall puncture: no difference. |
| **Artikel** | **Study type** | **Method** | | | | **Results** | |  |
| Jung et al. (55) | Delphi method | 15 Physician specialists. **Delphi study:** 3 blinded delphi-rounds. | | | | 16-item procedure performance checklist. | |  |
| Primdahl et al (54) | Delphi method | 14 specialists.  Delphi rounds: 3 delphi-rounds, starting with 9 Items. After 3 delphi-rounds 8 items were left. | | | | 5 point likert-scale including 8 items to evaluating the competency of USG-PVC placement | |  |
|  | | |  |  |  |  |  |  |

DIVA: difficult intravenous access
PVC: peripheral vein catheter
US: ultrasound

USG-PVC: Ultrasound guided peripheral vein catheter.
ED: Emergency Department
CVC: central vein catheter.
EM: emergency medicine
OSCE: objective structured clinical examination

SD: standard deviation
